# Health Sufficiency Indicators for Pandemic Monitoring

**DOI:** 10.3390/ijerph18105358

**Published:** 2021-05-18

**Authors:** Javier M. Moguerza, Salvador Perelló Oliver, Isaac Martín de Diego, Víctor Aceña, Carmen Lancho, Marina Cuesta, César González Fernández

**Affiliations:** 1 Data Science Laboratory, Rey Juan Carlos University, 28933 Móstoles, Spain; javier.moguerza@urjc.es (J.M.M.); isaac.martin@urjc.es (I.M.d.D.); victor.acena@urjc.es (V.A.); carmen.lancho@urjc.es (C.L.); cesar.gonzalezf@urjc.es (C.G.F.); 2 Young Academy of Spain, 28046 Madrid, Spain; 3 Methaodos.org, Rey Juan Carlos University, 28933 Móstoles, Spain; salvador.perello@urjc.es; 4 Madox Viajes, Jof Associates int S.L.U., 28939 Arroyomolinos, Spain

**Keywords:** indicators, COVID-19, fusion of information, health, time series

## Abstract

The outbreak of the COVID-19 disease, spreading all around the world and causing a worldwide pandemic, has lead to the collapse of the health systems of the most affected countries. Due to the ease of transmission, early prevention measures are proved to be fundamental to control the pandemic and, hence, the saturation of the health systems. Given the difficulty of obtaining characteristics of these systems of different countries and regions, it is necessary to define indicators based on basic information that enable the assessment of the evolution of the impact of a disease in a health system along with fair comparisons among different ones. This present paper introduces the Health Sufficiency Indicator (HSI), in its accumulated and daily versions. This indicator measures the additional pressure that a health care system has to deal with due to a pandemic. Hence, it allows to evaluate the capacity of a health system to give response to the corresponding needs arising from a pandemic and to compare the evolution of the disease among different regions. In addition, the Potential Occupancy Ratio (POR) in both its hospital ward bed and ICU bed versions is here introduced to asses the impact of the pandemic in the capacity of hospitals. These indicators and other well-known ones are applied to track the evolution of the impact of the disease on the Spanish health system during the first wave of the pandemic, both on national and regional levels. An international comparison among the most affected countries is also performed.

## 1. Introduction

Data, like respirators, save lives, but only if used properly. In economics, health and many other aspects of our lives, indicators and indexes are used to capture a complex reality in numbers. Thanks to these indicators, it is possible to study the evolution of many phenomena and make valid comparisons. In the same way that indexes such as the Gross Domestic Product (GDP), the unemployment rate or the risk premium are used to define and adjust economic measures, health indicators are used to design, evaluate and refine public health policies. Moreover, these indicators enable to know the diverse and changing reality of each territory and, therefore, to choose with precision the moment and location to implement, for example, the different phases of de-escalation of the containment measures aimed at stopping the spread of the Sars-CoV-2 virus.

The evolution of the COVID-19 pandemic is being followed with great attention. National, regional, local governments and the media report different graphics every day, generally divided into three categories, namely, diagnosed cases, recovered cases and deaths. In addition, data from different countries and regions are often compared. However, the conclusions of this exercise have important shortcomings. First, the data for each country depends largely on its size, density and demographic structure. Furthermore, each country uses different criteria to define each of the above-mentioned categories. Finally, in many countries very few diagnostic tests have been performed, so data are unreliable. For these reasons, comparing raw data is not a very useful strategy and it is generating confusion and distrust in the public opinion. However, this does not mean that fair comparisons cannot be made or that these figures are not valid. A useful and simple strategy is to consider these data as mere samples of the state of the pandemic, rather than as an accurate measure of the effects of the pandemic. Through this methodology, the tools of modern statistics can be used to design indexes that capture a complicated and changing reality, such as the human development index or the competitiveness index of a country.

The indicators are instruments that make it possible to reflect a complex reality referring to a given moment or time interval. They are projected in statistical series or any form of measurement that facilitates understanding the situation and direction of specific objectives and goals, as well as evaluating specific programs and determining their impact.

The indicators have two main purposes. On the one hand, they serve to describe the magnitude of a phenomenon so that comparisons between different territories can be made. On the other hand, they enable to evaluate the evolution of the phenomenon itself. These tools are particularly useful for the authorities, since they allow them to evaluate the effectiveness of their public policies and to monitor the impact of the measures they have executed as well as to compare them with those implemented in other countries. For all this to be possible, it is necessary to design indicators that describe the phenomenon to be studied in an appropriate manner, without bias or uncertainty.

The first thing that it should be required for a good indicator to be used to compare among different territories is to behave in a similar way in all of them. Another ideal requirement is that its range of possible values is the same regardless of the sample size. This ties in with the convenience of the indicator being limited in its values so that the end of the phenomenon can be anticipated. This is a feature that raw data do not have since, in this case, the final number of diagnosed cases, recovered and deaths are unknown.

It is clear that ineffective care can lead to unnecessarily poor outcomes for patients that are directly affected by the virus, measured either in terms of their improved health or in their wider satisfaction with the health system. More generally, inefficiency somewhere in the health system is likely to deny treatment and health improvements to patients who would otherwise have received treatment if resources had been better managed, especially in situations associated with a pandemic. Health system efficiency metrics should be useful for the following purposes: facilitating the analysis of policies; identifying the best practices; and detecting areas of the health system that are not performing as well as desired and that could potentially benefit from reforms [1].

This paper presents an indicator, the Health Sufficiency Indicator (HSI) in its accumulated and daily versions. The HSI gives a measure of the cases that leave the health system in relation to the total number of diagnosed cases at any given time. The HSI meets the ideal characteristics previously mentioned and allows to assess where each local region or country stands in the fight against a pandemic. Low values of the HSI will indicate a lack of resources in the health system (and therefore additional resources should be provided), while high values of the HSI will evidence that the system has sufficient means to take care of patients. In other words, the HSI measures the capacity of territories to respond to a disease. During the first wave of the COVID-19 pandemic, the Spanish Ministry of Science and Innovation have received daily reports from the Young Academy of Spain with this indicator [2,3]. The present work includes and extends the very preliminary results in [4]. The first block analyses the data from a specific region of Spain which, due to its size, could represent a situation similar to that of a hospital. In the next block, national data are analyzed both at a global and regional level. The last block presents a comparative study of the evolution in Spain in relation to the countries most affected by the pandemic. The analysis is based on the monitoring of the evolution of both accumulates and daily indicators, comprising all patients treated (recovered and dead) by the respective health systems since the beginning of the crisis caused by the SARS-CoV-2 virus. Section 5 includes a brief discussion. Section 6 concludes.

The present article is organized as follows. Section 2 provides an overview of some of the most recent contributions to measure the adequacy of a sanitary system, as well as to monitor pandemic situations. The definitions of other indexes and indicators, and the characteristics of the proposed HSI are explained in Section 3. The experiments, focused on the COVID-19 pandemic, are presented in Section 4. They are developed around three blocks: Local, National and International.

## 2. Pandemic Monitoring

The society deserves the most complete and accurate data available for COVID-19 pandemic in any region or country of the world. However, in many cases, none official source is providing this data. Thus, universities and scientists around the world have been forced to create and estimate new indicators to evaluate and to monitor the consequences of the pandemic. For instance, using data from [5], the Johns Hopkins University supplies an estimator of the US Hospitalization Rate [6]. Some local or national governments have reported cases and deaths caused by the pandemic. Nevertheless, the association between death and disease has not been homogeneous across countries or even within countries. The Incidence Rate, number of cases per million persons, has been used to compare different countries [7]. Most international research has been also forced to use estimators for the number of recovered cases, based on local media reports, and state and local reporting when available [6].

There is a need to create a monitoring and evaluation framework that provides a set of standardised indicators to guide regional, national and international governments in the implementation and effects of COVID-19 pandemic response activities. The framework presented for the EU/EEA and the UK, uses indicators for a variety of the key aspects in the COVID-19 preparedness, prevention and control activities. This offers a guidance to countries on how to collect and analyse data for the suggested indicators [8]. One of the pillars of this framework is to preserve critical health services and systems with the specific objective of monitoring the potential impact of the pandemic on the delivery of care in hospitals and the health system. To achieve this aim the following indicators are proposed in the literature:

- Occupancy rate of total ICU beds (overall and for COVID-19 patients).
- Number of registered visits to primary care.
- Measles incidence and proportion of all cases among unvaccinated children whose first dose of the MMR (Measles, Mumps, Rubella) vaccine was during the COVID-19 pandemic.
- Diphtheria-tetanus-pertussis (DTP)-3 vaccination coverage in children under 12 months of age.

Therefore, the main indicator of the health system’s capacity to treat critically ill patients is the occupancy rate of the total number of ICU beds. The total number of available ICU beds, expressed by 100,000 population, is also an indicator of the health system’s ICU base capacity. This indicator should be daily measured at national and regional levels. In order to obtain this indicator, it should be reported by hospitals.

Another relevant indicator is the number of registered visits to the primary care. This indicator could reveal a high use of the health system in periods of epidemic exacerbation or a underuse due to the stay-at-home recommendations or to the fear of patients to use primary care services.

The other two indicators measure the capacity of the health system to guarantee and support other critical health preventive activities during the outbreak.

Several epidemiological indicators of the impact of the COVID-19 epidemic are used in the official reports. Their strengths and limitations are presented in [9]. The most commonly used indicator is the case fatality proportion defined as the proportion of infected cases that die due to the disease.

In addition, indicators for the dynamic of the spread of an infection in a population are presented in [10]:

- The base case reproduction number, $R_{0}$: represents the average number of people to whom one infected person transmits the infection, during their infectious period, in a population with no immunity and no specific infection control measures.
- The effective reproduction number, R(t): represents the average number of people at time *t* to whom an infected person transmits the infection during his or her infectious period, in a population without immunity and without specific infection control measures.

Notice that R(t) is an important indicator during the course of the dissemination of an infection in a population, as it takes into account the effect of control measures and the rate of spread of infection [11]. On the one hand, when R(t)>1, the number of new cases will increase further. On the other hand, when R(t)<1, the infection will start to come to an end.

A high number of papers using the Infection Fatality Rate (IFR) are presented (see, for instance, [12,13,14]). The IFR estimates the fatality rate in all the infected patients, including those with the disease detected (i.e., confirmed cases) and those with the disease undetected (asymptomatic and not tested group). Notice the differences between the IFR and the Case Fatality Rate (CFR), that is the number of reported deaths per number of reported cases. In [15], a comparison of the incidence rate and the CFR for 154 countries is presented. In [16], an adjusted version of CFR is presented to account for delay to outcome, where case and death incidence data are used to estimate the number of cases with known outcomes (death or recovery).

## 3. Materials and Methods

In the present work, the Health Sufficiency Indicator (HSI) is proposed in both its daily (dHSI) and accumulated (aHSI) versions. Both indicators measure the additional pressure that a health care system has to deal with due to a pandemic. Thus, the proposed indicators allow to evaluate the capacity of local or national health systems to give response to the corresponding needs derived from a pandemic. The dHSI and aHSI also ease a fair comparison of the evolution of the pandemic among different countries, which can be sometimes confusing as a consequence of the different data gathering protocols.

Two other complementary indicators are here defined: the Hospital Potential Occupancy Ratio (hPOR) and the ICU Potential Occupancy Ratio (icuPOR). They measure the potential bed occupancy of hospitals during the pandemic due to the disease. Thus, they enable to detect possible situations of hospital ward beds saturation or ICU beds occupancy saturation that might be about to happen, when no accurate data about hospital occupancy is being collected during the pandemic.

Next, the definition of the proposed indicators and other helpful variables to track the evolution of a pandemic are presented. The fusion of a number of indicators, probably from different sources of information, are needed in order to monitor health sufficiency at local and national levels. The main properties of the proposed indicators are discussed.

### 3.1. Definitions

The following variables contains the basic information related to the disease of a pandemic in order to track its evolution. They are generally provided by official organisations or institutions of a country and comprise accumulated information.

- $Confirmed_{t}$: Number of accumulated cases confirmed through a medical test to date *t*.
- $Deaths_{t}$: Number of accumulated deaths of previously confirmed patients to date *t*.
- $Recovered_{t}$: Number of accumulated recoveries of previously confirmed patients to date *t*.

Other helpful information to track the epidemic that might be available by official sources is:

- $HospitalBedOccupancy_{t}$: Number of occupied beds in hospital ward due to the disease on date *t*
- $ICUBedOccupancy_{t}$: Number of occupied beds in the ICUs of hospitals due to the disease on date *t*.
- $HospitalDischarge_{t}$: Number of hospital discharges of previously confirmed cases on date *t*.
- $PrimaryCareDischarge_{t}$: Number of discharges in the primary health care system of previously confirmed cases on date *t*.
- HospitalBedCapacity: Total number of beds in hospital wards, including beds in the Intensive Care Units (ICUs).
- ICUBedCapacity: Total number of beds in ICUs.

From the variables presented above, some other variables can be computed when they are not provided by official organisations or institutions:

- $Solved_{t}=Recovered_{t}+Deaths_{t}$: the accumulated number of confirmed cases with known outcomes, that is, those cases that have been solved to date *t* (either due to a recovery or death).
- $DailyConfirmed_{t}=Confirmed_{t}-Confirmed_{t-1}$, number of new daily confirmed cases on date *t*.
- $DailyDeaths_{t}=Deaths_{t}-Deaths_{t-1}$: number of new deaths on date *t*.
- $DailyRecovered_{t}=Recovered_{t}-Recovered_{t-1}$: number of new recoveries on date *t*.
- $DailySolved_{t}=DailyRecovered_{t}+DailyDeaths_{t}$: number of cases that have been solved on date *t*.
- $ActiveCases_{t}=Confirmed_{t}-Deaths_{t}-Recovered_{t}$: number of active confirmed cases of the disease at the end of date *t*. That is, number of patients with the disease at the end of date *t*. It comprises the count at the end of date *t* since the counting include the new cases on date *t* but not the new deaths and neither the recovered.
- $PreActiveCases_{t}=ActiveCases_{t-1}$: number of active confirmed cases of the disease at the beginning of date *t*. That is, number of patients with the disease at the beginning of date *t*.

Furthermore, through the following percentage variables, the basic information related to the disease of a pandemic can be represented in a relative way. Thus, fair comparisons among different health systems can be performed.

- $DeathsPercentage_{t}=\frac{Deaths_{t}}{Confirmed_{t}}\times 100$: percentage of accumulated deaths to date *t*.
- $RecoveredPercentage_{t}=\frac{Recovered_{t}}{Confirmed_{t}}\times 100$: percentage of accumulated recoveries to date *t*.

### 3.2. Health Sufficiency Indicators

In this work, the Health Sufficiency Indicators are defined to track the evolution of a pandemic from the point of view of the sufficiency of a healthcare system. Two versions are here proposed: the accumulated version and the daily one. The former one gives a global perspective of the pandemic while the second provide specific information of the corresponding date.

The Accumulated Health Sufficiency Index (aHSI) measures the accumulated stress of a healthcare system due to the pandemic. For a day *t*, the aHSI is defined as follows:

(1) $$aHSI_{t}=100\times \frac{Solved_{t}}{Confirmed_{t}}\;,$$

Notice that, aHSI varies between 0% and 100%. The greater the aHSI is for a system, the more de-stressed the situation is. High values of aHSI reveal that the system has the adequate sufficiency to treat all the patients and related problems that are derived from a pandemic. Thus, values close to 100% are related to a controlled situation of the pandemic.

The Daily Health Sufficiency Index (dHSI) measure the daily stress of a healthcare system due to the pandemic. In general terms, for a day *t*, dHSI is defined as follows:

(2) $$dHSI_{t}=\frac{DailySolved_{t}}{DailyConfirmed_{t}}\;.$$

However, there are some exceptions in its definition:

- If $DailySolved_{t}=0$ but $DailyConfirmed_{t}\neq 0$, then
  $$dHSI_{t}=\frac{1}{DailyConfirmed_{t}+1}$$
- If $DailySolved_{t}\neq 0$ but $DailyConfirmed_{t}=0$, then
  $$dHSI_{t}=DailySolved_{t}+1$$
- If $DailySolved_{t}=0$ and $DailyConfirmed_{t}=0$, then
  $$dHSI_{t}=1$$

Notice that, $dHSI_{t}$ is greater or equal to 0, without an upper limit. The greater the index is, the more de-stressed is the situation. In addition, several days with consecutive values under 1 imply a possible temporal saturation of the healthcare system. On the contrary, $dHSI_{t}$ values above 1 for a long time evince better sufficiency levels.

#### 3.2.1. Properties of the HSI

In order to consider that a health system is starting to get stabilized during a pandemic is necessary to consider both indicators at the same time. Notice that a stable situation is achieved when the $dHSI_{t}$ sustainedly keeps values above 1, and a prolonged growing tendency of the $aHSI_{t}$ is observed. The following propositions are valid when there are daily solved and confirmed cases. That is, the initial (with no daily solved cases) and the final (with no new confirmed cases) stages are not considered.

**Proposition** **1.** *The daily Health Sufficiency Indicator on a given day t is greater than the accumulated one divided by 100 on the previous day, if and only if the accumulated Health Sufficiency Indicator on that day increases regarding the day before. That is:*

(3) $$aHSI_{t}>aHSI_{t-1}⟺dHSI_{t}>\frac{aHSI_{t-1}}{100}$$

**Proof.**  To prove the previous relation of Equation (3), lets first point out that by definition:

$$\begin{array}{cc} aHSI_{t-1} & =100\times \frac{Solved_{t-1}}{Confirmed_{t-1}}\\ dHSI_{t} & =\frac{DailySolved_{t}}{DailyConfirmed_{t}} \end{array}$$

Thus, $aHSI_{t}$ can be also expressed as

$$aHSI_{t}=100\times \frac{Solved_{t-1}+DailySolved_{t}}{Confirmed_{t-1}+DailyConfirmed_{t}}$$

Then,

$$\begin{array}{cc} \; & aHSI_{t}>aHSI_{t-1}\\ ⟺ & \frac{Solved_{t-1}+DailySolved_{t}}{Confirmed_{t-1}+DailyConfirmed_{t}}>\frac{Solved_{t-1}}{Confirmed_{t-1}}\\ ⟺ & Confirmed_{t-1}\times \left(Solved_{t-1}+DailySolved_{t}\right)>\\ \; & Solved_{t-1}\times \left(Confirmed_{t-1}+DailyConfirmed_{t}\right)\\ ⟺ & Confirmed_{t-1}\times DailySolved_{t}>Solved_{t-1}\times DailyConfirmed_{t}\\ ⟺ & \frac{DailySolved_{t}}{DailyConfirmed_{t}}>\frac{Solved_{t-1}}{Confirmed_{t-1}}\\ ⟺ & dHSI_{t}>\frac{aHSI_{t-1}}{100} \end{array}$$

□

**Proposition** **2.** *The daily Health Sufficiency Indicator is higher than 1 if and only if there is an increment on the number of active cases. That is:*

(4) $$dHSI_{t}>1⟺ActiveCases_{t-1}>ActiveCases_{t}$$

**Proof.**  It can be proven as follows:

(5) $$\begin{array}{cc} dHSI_{t}>1⟺ & \frac{DailySolved_{t}}{DailyConfirmed_{t}}>1\\ \;\;\;\;\;⟺ & DailySolved_{t}>DailyConfirmed_{t}\\ \;\;\;\;\;⟺ & DailySolved_{t}-DailyConfirmed_{t}>0 \end{array}$$

Adding the term $Confirmed_{t-1}-Solved_{t-1}$ to each side of the inequality:

(6) $$\begin{array}{cc} & DailySolved_{t}-DailyConfirmed_{t}>0\\ \; & ⟺Confirmed_{t-1}-Solved_{t-1}+DailySolved_{t}-\\ \; & DailyConfirmed_{t}>Confirmed_{t-1}-Solved_{t-1}\\ \; & ⟺Confirmed_{t-1}-Solved_{t-1}>(Confirmed_{t-1}+\\ \; & DailyConfirmed_{t})-\left(Solved_{t-1}+DailySolved_{t}\right)\\ \; & ⟺Confirmed_{t-1}-Solved_{t-1}>Confirmed_{t}-Solved_{t}\\ \; & ⟺ActiveCases_{t-1}>ActiceCases_{t} \end{array}$$

□

#### 3.2.2. Interpretation of the HSI

The dHSI measures the capacity of a health care system to face a disease on a determined date. Thus, the dHSI is measuring the stress suffered by a system on a short period of time. In contrast, the aHSI encompasses the sufficiency that the health system is having along a more extended period of time. That is, the aHSI measures the stress suffered by a system for a long period of time. Consequently, it is of great interest to study both indicators jointly during a pandemic.

Through the dHSI it is possible to detect the occasional outbreaks of a disease or the beginning of pandemic, where the flow of new patients compared to the solved one is much higher. However, aHSI enables to analyse if the disease is being controlled by the health system.

Concerning the examination of the evolution of the pandemic on a daily basis, three different scenarios for the corresponding health system can be established. These scenarios are defined based on the values of the daily and accumulated versions of the HSI indicators each day *t*. This enables an early detection of a deterioration in the pandemic progression. Thus:

- $dHSI_{t}>1$ and $dHSI_{t}>\frac{aHSI_{t-1}}{100}$⟶ good scenario.
- $\frac{aHSI_{t-1}}{100}<dHSI_{t}<1$⟶ ordinary scenario.
- $dHSI_{t}>\frac{aHSI_{t-1}}{100}$⟶ bad scenario.

During a pandemic, low values of the aHSI are expected, specially at the very beginning of it. When the aHSI presents low values the impact of disease in the population is not controlled and it is necessary to take urgent actions to enlarge the capacity of the system. Those actions include massive hiring of medical personnel or the set up of additional public spaced to treat patients.

As stated in Section 3.2, aHSI values close to 100% reveal that a disease is controlled in the population since there is a balance between the confirmed cases and the solved ones. However, a health care system could be collapsed when an aHSI close to 100% is observed. When that situation occurs, then the health system has insufficient resources to face the needs of the population and it is necessary to take permanent actions to strengthen it. On a totally opposite situation, if a health case system is not even saturated with low aHSI values, then the health care system has much more resources than needed, which might not be economically adequate.

### 3.3. Potential Occupancy Ratios

The Potential Occupancy Ratio (POR) is here defined for two clases of hospital beds: common hospital ward beds and ICU beds. The first ratio is named as Hospital Potential Occupancy Ratio (hPOR) and the second as ICU Potential Occupancy Ratio (icuPOR). They are defined to evaluate the progress of the pandemic from a point of view of the capacity of hospitals.

The hPOR measures the ratio of active cases per ward beds and ICU beds in hospitals on date *t*:

$$hPOR_{t}=\frac{PreActiveCases_{t}}{HospitalBedCapacity}$$

If $hPOR_{t}<1$, then there is a full potential healthcare assistance, since there is a hospital bed for each active patient.

The icuPOR measures the ratio of active cases per common ICU beds in hospitals on date *t*:

$$icuPOR_{t}=\frac{PreActiveCases_{t}}{ICUBedCapacity}$$

As for the hPOR, the desired values of icuPOR are under 1, since in that case, there would be a ICU bed for every critical patient with the disease.

Both previous indicators are useful when no data about hospital occupancy is being collected during the pandemic to evaluate the hospital saturation level and to anticipate a possible collapse.

## 4. Results

The health systems of most of the countries affected by the COVID-19 pandemic have collapsed due to the extremely high incidence. On the one hand, given the high speed of spread of this epidemic disease, in many cases, local and national authorities have not had the appropriate information collection and processing tools to adequately monitor the pandemic. On the other hand, the epidemiological information is so vast and heterogeneous that effective indicators are needed to take benefit of it and to draw unbiased conclusions.

The indexes and indicators that have been previously presented in Section 3.1 are here applied to the track COVID-19 pandemic. Thus, the fusion of indicators from different sources of information are considered, related to three different levels of generalization. In a local case, specific and accurate information should be provided. This case could be compared with the information obtained from hospital sources, that is, high-quality data are collected very close to the source of information. The more local the study is, the more data is prone to be available. In a country case, a loss of data quality is expected as data is often aggregated from local information. In an international case, general and easily comparable indicators should be considered.

Next, the proposed indicators are used to monitor the COVID-19 pandemic in those three levels of generalization. First, a local case application is performed in the Spanish region of La Rioja in Section 4.1. Then, a more general study over the whole health system of Spain is carried out in Section 4.2. Finally, the evolution of the health system of the most affected international countries are compared in Section 4.3. In these three cases of application, only the first wave of the COVID-19 pandemic is studied. The HSI indicators are useful to detect early stages of a disease and to track their evolution until a control situation is achieved. Hence, at the end of a determined wave, when the health care system has none or very low additional pressure due to the pandemic and the aHSI is very close to 100%, all the indicators presented above should be reset to 0 in order to correctly track the next wave. This implies that the analysis of consecutive waves of the COVID-19 pandemic would be the same as the analysis of the first wave.

### 4.1. Local Case

The evolution of COVID-19 pandemic is tracked and studied in the autonomous community of La Rioja in Spain. This is a local case application since data related to COVID-19 comprises only a small region with a population of about 320,000 people. This community has 20 health centres, 1 hospital with 17 ICU beds (at the beginning of the pandemic) and 3 other hospitals (without ICU beds). It is necessary to clarify that each Spanish autonomous community manages its own health system. All the variables containing official data about COVID-19 are available for La Rioja in sources [17,18]. Besides, bed capacity data in hospitals in La Rioja is available in [19,20]. Thus, in this case, all the indicators presented in Section 3.1 can be estimated for the health care system of La Rioja. The presented results focus on the daily and accumulated versions of the HSI, as well as on the hPOR and icuPOR.

**Figure 1:**
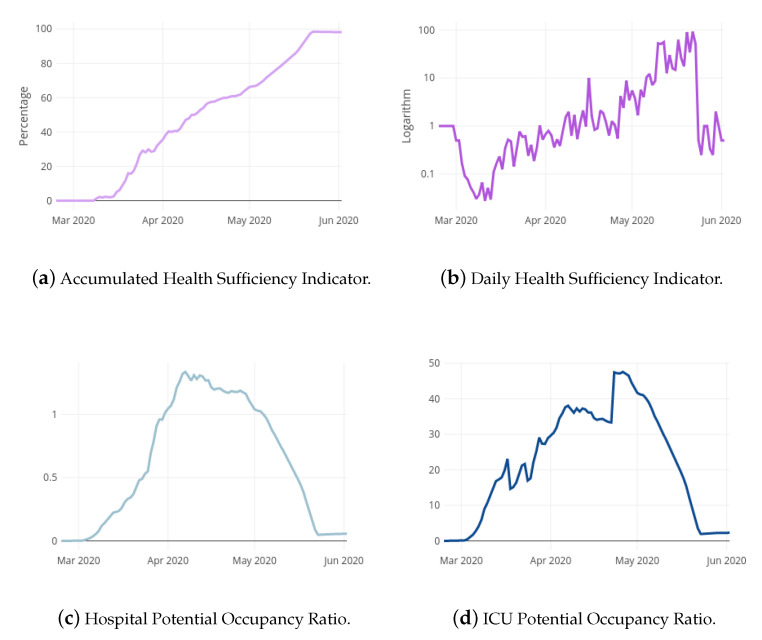
Health Sufficiency Indicators and Potential Occupancy Ratios for the local case.

The impact of COVID-19 pandemic in La Rioja is here studied from 24 February 2020 to 2 June 2020. Figure 1a,b show the evolution of the aHSI and dHSI indicators, respectively, in La Rioja. The aHSI has followed an increasing tendency during the whole COVID-19 outbreak, achieving values greater than 80% on middle May. The dHSI consistently surpassed value 1 on middle April. Figure 1c,d present the evolution of the hPOR and icuPOR ratios in La Rioja, respectively. The hPOR under-passed value 1 at the beginning of May and the icuPOR ceaselessly turned into a decreasing trend at the end of April. The four graphics of Figure 1 reveal altogether that the pandemic COVID-19 could be considered as controlled approximately on middle May.

One of the major problems derived from the COVID-19 pandemic has been the saturation and collapse of the health systems and, in particular, of the hospital systems. This has aggravated the number of deaths due to the disease. The anticipation of the incidence of the pandemic in the population and an adequate management of the available resources are the key elements to prevent the collapse in the health care systems. Hence, it is extremely urgent, interesting and useful to design mathematical models that, using all the information described in Section 3.1, are capable of predicting peaks of saturation in the sanitary resources in the following days.

In the present paper, the predicting task in La Rioja is centred on two hospital resources: the hospital ward beds and the hospital ICU beds. That is, the target variables are $HospitalBedOccupancy_{t}$ and $ICUBedOccupancy_{t}$. With the prediction of the hospital ward and ICU beds occupancy and with the corresponding total capacities, it is possible to anticipate peaks of saturation in the hospital bed occupancy in the following days and take the necessary actions to mitigate it.

**Figure 2:**
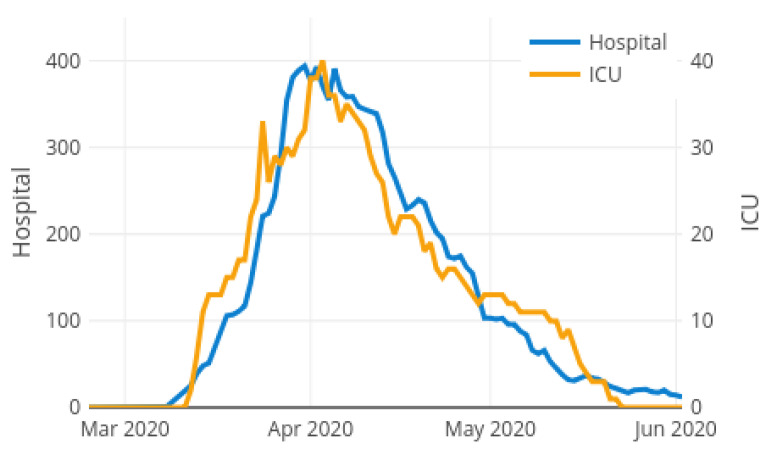
Target variables for the local case model: Hospital ward and ICU bed occupancy.

Figure 2 shows the observed curves of variables $HospitalBedOccupancy_{t}$ and $ICUBedOccupancy_{t}$ during the COVID-19 pandemic in La Rioja from from 24 February 2020 to 2 June 2020. Both target curves can be divided into three different phases: increasing phase, valley phase and decreasing phase. During the increasing phase, the number of hospitalised patient grows rapidly, getting stabilized and reaching its maximum in the valley phase. The number of occupied beds due to the pandemic starts to lower in the decreasing phase, until it is close to zero when the pandemic is controlled. It is pursued an anticipation of 4 days in the detection of possible collapse of hospital ward and ICU beds. Thus, all the necessary logistical operations can be implemented both in the ICU and in the common hospital wards to be able to face the epidemic while avoiding collapse.

**Figure 3:**
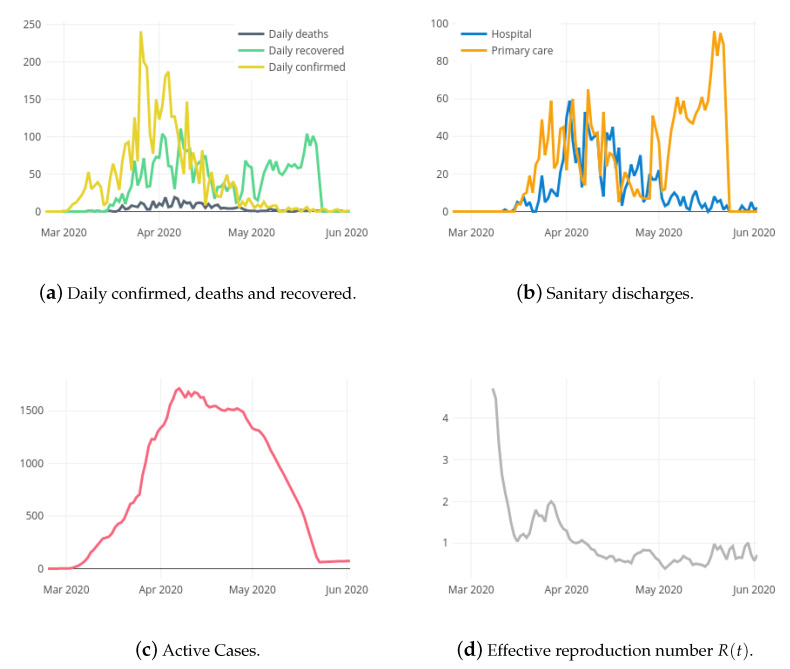
Exogenous variables for the local case model.

$DailyConfirmed_{t}$, $DailyDeaths_{t}$, $DailyRecovered_{t}$, $HospitalDischarge_{t}$, PrimaryCare$Discharge_{t}$, $ActiveCases_{t}$, $R_{t}$, $aHSI_{t}$, $dHSI_{t}$, $hPOR_{t}$ and $icuPOR_{t}$ are included as exogenous variables into the predictive models for both target variables. These variables might help in the explanatory task and in the prediction accuracy. The EpiEstim package of R [21] is used for the estimation of R(t) where it is considered the absolute daily increase of cases and deaths in the last seven days. Following [22], an average time between infections of 4.7 days with a standard deviation of 2.9 is assumed. Figure 1 and Figure 3 shows the curves of the exogenous variables during the COVID-19 pandemic in La Rioja. Notice that the relation between the exogenous variables and the corresponding target may vary depending on the phase of the curve.

In this paper, a prediction method with two different forecast horizons (1 and 4 days) is proposed. This method is useful to anticipate collapse situations in the hospital wards and ICU beds. It consists of the following three steps:

1. Automatic detection of the actual phase: increase, decrease or valley. The system is inspired by the Adaptive Windowing (ADWIN) method for change detection [23] and the Trent Segmentation Algorithm (TSA) [24], a linear segmentation algorithm based on the idea of looking for feature points where extreme changes on the data trend occurs. The ADWIN method uses two adaptive windows for the distribution drift, which leads to a slow detection of gradual changes. To solve this handicap, three fixed windows are selected, in order to achieve the early detection of the curve shift. In each window, a simple regression linear model is adjusted, where the slope of each line represents the growth rate of the variable response (hospital wards or ICU beds), on its current window. The slopes represent the different phases, according to their value: slope greater than zero corresponds to increase phase, less than zero corresponds to decrease, and finally zero corresponds to valley phase. For each slope of the three windows, we have one local prediction, and the final phase estimation is obtained by voting. In the case of three different phase local prediction, the middle window corresponding slope is the selected global estimation. The three windows length are based on the incubation period of COVID-19 [25]. Therefore, the lengths of big, middle and small windows are 10, 5 and 3 days, respectively.
2. Automatic selection of the exogenous variables that are correlated with the corresponding target and so, might help in the prediction accuracy. In this step, the linear correlation among all exogenous variables differentiated and the target are estimated. First, all the variables which are not significantly correlated (using α=0.05) are removed. Second, the correlation among those remaining explanatory variables is analysed in order to avoid co-linearity. If some of the variables present a significant linear relationship, the one showing a higher correlation with the rest of variables is deleted. This is repeated until there is no co-linearity among the final selected exogenous variables.
3. Training of a Dynamic ARIMA model [26] with the selected explanatory variables as exogenous variables. For each prediction date, the best ARIMA model is automatically fitted. Hence, the ARIMA parameters are dynamic along the time. For each forecast horizon Hz=1 and Hz=4, the selected explanatory variables from the previous step are included into each fitted ARIMA model with lags Hz and Hz+1. One exogenous variable is created for each explanatory variable and each lag order. A backward selection method is applied to the set of regressor variables so that only those statistically significant for the fitted ARIMA model remain.

**Figure 4:**
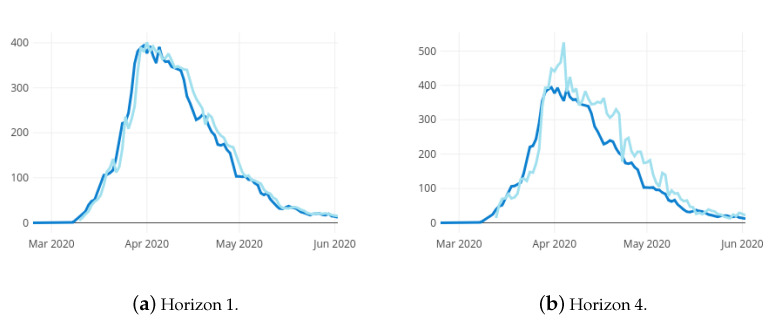
Observed values (dark blue) and prediction results (light blue) for the hospital ward bed occupancy in La Rioja with 2 different forecasting horizons.

Figure 4a,b show the prediction results estimated for the hospital ward bed occupancy ($HospitalBedOccupancy_{t}$) with forecasting horizons 1 and 4, respectively, and the real observed bed occupancy. Due to the necessary size of training set, the first possible predicted date is 10 March 2020. It can be observed that, as the prediction horizon is farther, the prediction error increases. As expected, the error increases along with the prediction horizon. The Root Mean Square Error (RMSE) shifts from 12.80±16.39 for the one-day horizon up to 45.28±59.51 for the four-day horizon. Thus, the mean error for the one day horizon is about 13 beds and as it can be seen in Figure 4, the larger errors occur in the shift between increase and decrease phases (regarding that the RMSE penalises larger errors more). In addition, that prediction errors are often in excess, this implies that the model never underestimates the growth of the pandemic, which can be catastrophic.

**Figure 5:**
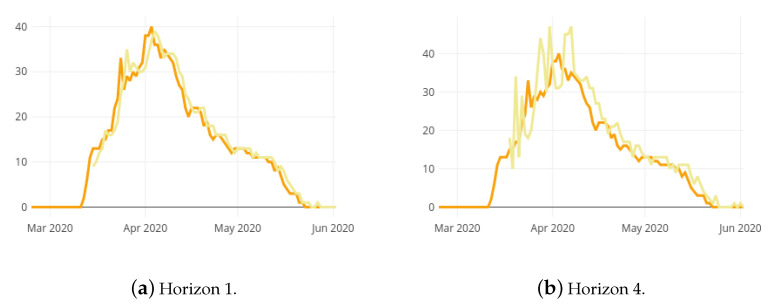
Observed values (dark orange) and prediction results (light orange) for the ICU bed occupancy in La Rioja with 2 different forecasting horizons.

Analogously, Figure 5a,b show the prediction results estimated for the hospital ICU bed occupancy ($ICUBedOccupancy_{t}$) with forecasting horizons 1 and 4, respectively, and the real observed data. The first possible predicted date is 15 March 2020. Similarly to the previous case, as the prediction horizon is farther, the prediction error increases. As in the hospital ward bed occupancy predictive model, the error increases along with the prediction horizon. The Root Mean Square Error (RMSE) shifts from 1.32±2.09 for the one-day horizon up to 5.06±7.60 for the four-day horizon. Thus, the mean error for the one day horizon is about one bed and as it can be seen in Figure 5, the larger errors result in the the increase phase. As in the previous case, that prediction errors are often in excess, which in the context of a pandemic, is a desirable scenario as opposed to underestimating growth.

### 4.2. Country Case

In this section, the indicators and indexes exposed in Section 3.1 are estimated and studied for the entire health care system of Spain during the COVID-19 pandemic. A comparison among the different regions of Spain is also performed here. In this country case, only the accumulated basic information and the hospital capacity variables enumerated in Section 3.1 are available for the corresponding health care systems. Bed capacity data in hospitals of each of the autonomous community of Spain is available in [19,20]. Official data about the COVID-19 pandemic is provided by the Spanish Ministry of Health in [27].

The evolution of the pandemic is here analysed from 16 March 2020 to 17 July 2020. The results exposed below have been daily published [28] in a report elaborated by the research groups Data Science Laboratory (DSLAB) [29] and Methaodos.org [30] of the Rey Juan Carlos University in Madrid (Spain), and the Academia Joven de España [31]. As stated in [2], these daily reports have been used as scientific support by the Government of Spain during the COVID-19 pandemic.

**Figure 6:**
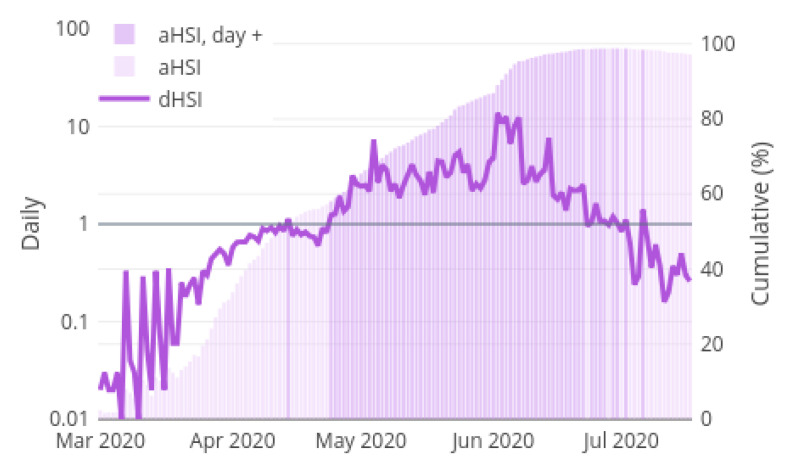
Accumulated Health Sufficiency Indicator and Daily Health Sufficiency Indicator in Spain.

Figure 6 shows the progression of the daily and accumulated Health Sufficiency Indicators (dHSI and aHSI) in whole health care system of Spain through out the pandemic. The bar chart with y-axis on the right presents the observed aHSI values each day. The dHSI appears as the line chart with the y-axis on the left. The aHSI is coloured in a dark purple when the corresponding value of the dHSI is greater than 1. These days are named as positive days, since the number of solved COVID-19 patients that day is higher than the new cases received into the health care system. As explained in Section 3.2, that is a sign of the health system recuperation in the pandemic. Figure 6 captures how the Spanish health care system suffered severe limitations handling the high amount of diagnosed cases at the beginning of the pandemic. At the end of April, the dHSI starts to sustainedly surpass value 1, which means that the pressure of the pandemic over the health system starts to lower, since resources are being daily released. It is not until one month later when, at the end of May, the aHSI exceed 80%. At the beginning of June, aHSI exceeds 95% and COVID-19 is controlled. However, the graphic show how in July the dHSI decreases significantly, under passing value 1. This causes the aHSI to lower as well. The dHSI behaviour in July is anticipating a second outbreak of the COVID-19 virus in Spain, where a lot of confirmed cases are being diagnosed but few of them are solved.

Since each of the autonomous communities in Spain manage its own health care system, it is of great interest to compare their evolution during the pandemic. This enables to apply different kind of measures against the propagation of COVID-19 to each of the autonomous communities according of their sufficiency level. In addition, this comparison allows to elaborate logistic plans to share resources between geographically close autonomous communities when needed.

In terms of aHSI, the 19 regions of Spain can be reduced to four representative patrons that comprise the behaviour during the COVID-19 pandemic of the rest of Spain: Castile and Leon, Autonomous City of Ceuta, Community of Madrid and Extremadura. This four medoids regions have been obtained through a time series clustering with a Partition Around Medoids (PAM) method to get the time series prototypes [32]. Castile and Leon represents Castile La mancha and Asturias. The Autonomous City of Ceuta encompasses the Autonomous City of Melilla and the Region of Murcia. The Community of Madrid is the representative patron of Canary Islands, Catalonia, Balearic Islands and La Rioja. Finally, Extremadura comprises the rest of Spain.

**Figure 7:**
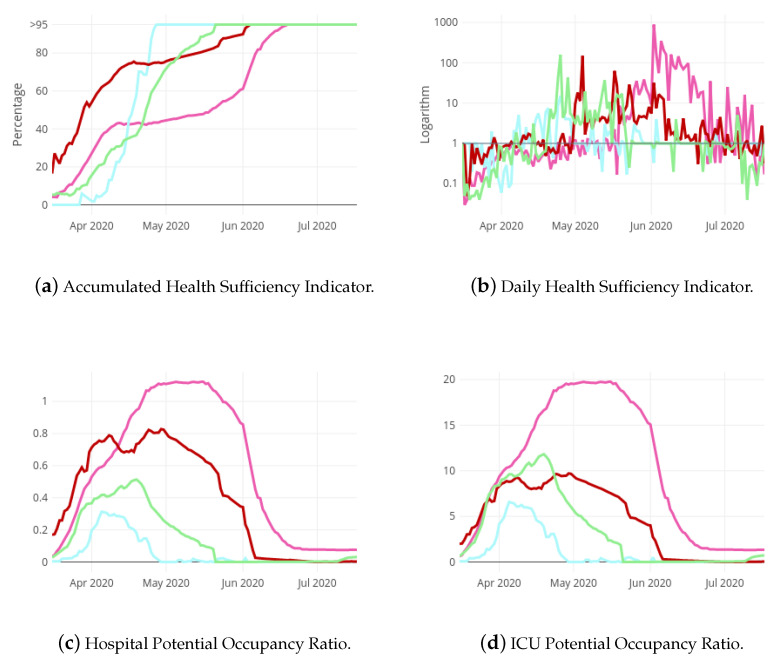
Evolution of Health Sufficiency Indicators and Potential Occupancy Ratios for four regions in Spain: Castile and Leon (pink), A. C. Ceuta (blue), C. Madrid (red) and Extremadura (green).

Figure 7 contains the curves of aHSI, dHSI, hPOR and icuPOR followed in each region. Notice that the dHSI is represented in a logarithm scale. The Autonomous City of Ceuta represents the less affected regions by the pandemic. In fact, its aHSI reached the 95% very early (Figure 7b) and its hPOR has never been greater than 0.4 (Figure 7c). On the contrary, the health systems in the regions represented by Castile and Leon have suffered the most due to the pandemic. Figure 7b shows a very plain aHSI curve for this region, with a slightly growing tendency. In addition, its dHSI has maintained values under 1 until middle May.

**Figure 8:**
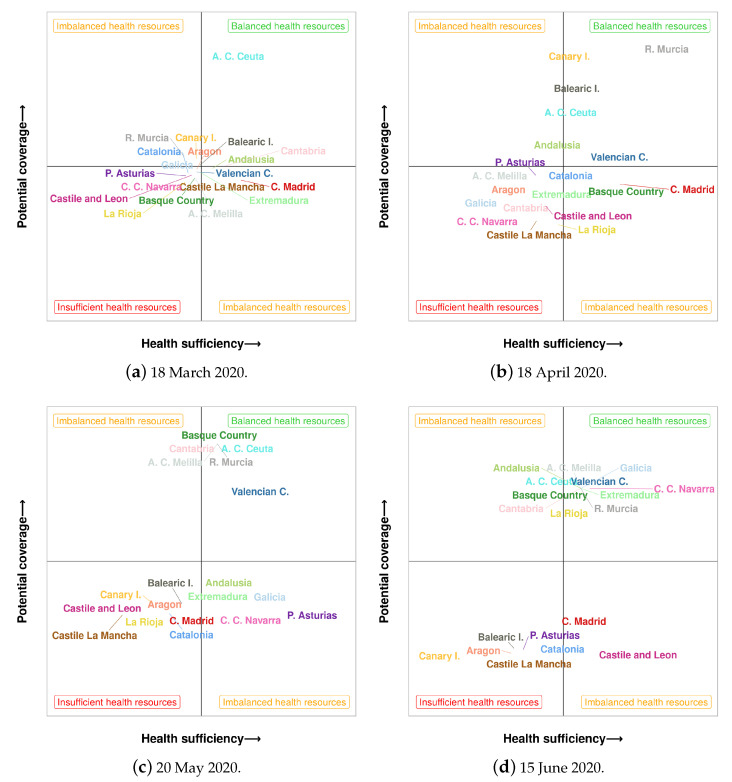
Evolution of the different regions of Spain in terms of health sufficiency and potential occupancy.

Figure 8 contains the evolution of the 19 regions of Spain in terms of the health sufficiency and the potential occupancy. Each subfigure represents a status chart, where the health sufficiency and the potential occupancy are crossed. The x-axis contains a measure of global health sufficiency computed as a combination of indicators aHSI and dHSI. Analogously, the y-axis contains a measure of global potential occupancy computed as a combination of hPOR and icuPOR. The resulting chart is divided into four quadrants that correspond to the following categories:

- Balanced health resources: high health sufficiency and high potential occupancy. This is the most desirable scenario.
- Imbalanced health resources: either high health sufficiency and low potential occupancy, or low health sufficiency and high potential occupancy.
- Insufficient health resources: low health sufficiency and low potential occupancy. This is the most unfavourable scenario.

Figure 8 comprises the status chart obtained for four dates along the pandemic: Figure 8a at the beginning of the pandemic in Spain, Figure 8b,c in the middle and, finally, Figure 8d at the middle of June. Hence, the evolution of the status of the 19 regions of Spain throughout this four dates can be analysed. Some communities such as Aragon or Castile La Mancha have never leaved the worst scenario of insufficient health resources. In turn, the autonomous city of Ceuta has stayed in the best scenario. Some other communities like C. C. Navarra or Extremadura have followed a desirable progression, achieving to locate in the best scenario on June, after going through the worst quadrant.

### 4.3. International Case

The last application example of the variables defined in Section 3.1 is a performance comparison of the most worldwide affected countries during the COVID-19 pandemic. The studied countries are Belgium, France, Germany, Italy, Russia, Spain, Switzerland and Turkey for the European continent; and Brazil, Canada, China, India, Iran, Peru, South Korea and the United States for the American and Asian continent. COVID-19 data is collected in [33], provided by the Johns Hopkins University.

**Figure 9:**
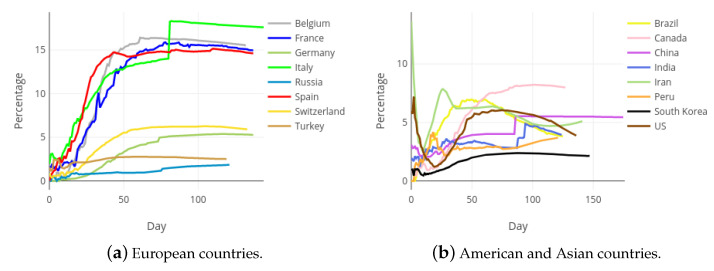
Accumulated deaths in most affected European, Asian and American countries.

**Figure 10:**
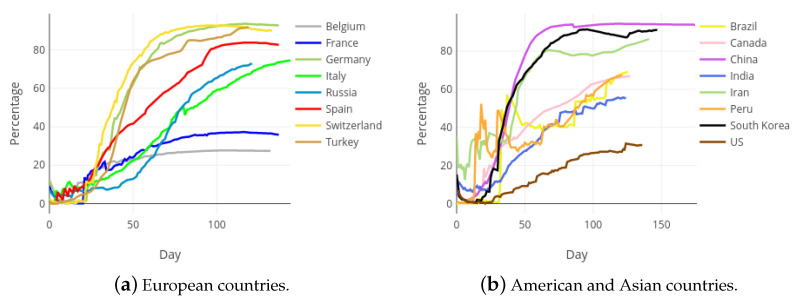
Accumulated recoveries in most affected European, Asian and American countries.

From the beginning of the COVID-19 outbreak, the impact of the pandemic in different countries has been assessed by directly comparing the figures reported by their authorities, mainly through the variables with basic information: confirmed cases, recovered patients and deaths. Figure 9 and Figure 10 contain the evolution of the accumulated number of COVID-19 deaths and the accumulated number of COVID-19 recoveries, respectively, in each of the mentioned countries. Notice that, pursuing a fair comparison, the x-axis of these figures correspond to the day of the pandemic, starting from day zero. Every graphic is truncated to the day 105 of the Spanish pandemic, which correspond to date 17 July 2020. The gross comparison derived from Figure 9 and Figure 10 presents important drawbacks, especially due to the different criterion used by countries to count those variables.

**Figure 11:**
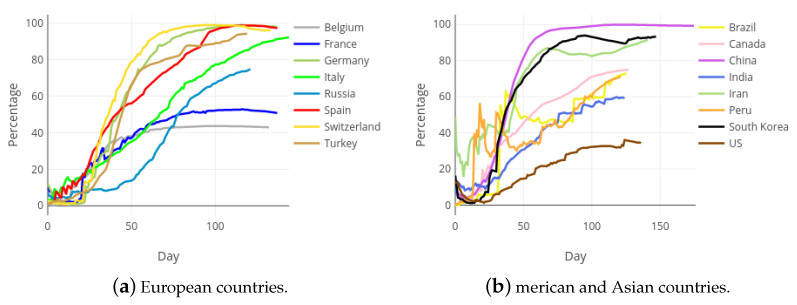
Accumulated Health Sufficiency Indicator in most affected European, Asian and American countries.

However, from a statistical point of view, the collected data can be considered as a sample, and not as a crude measurement of the reality. By using this strategy, it is possible to apply the techniques of modern statistics and estimate more useful indicators, as the accumulated and daily Health Sufficiency Indicators that are first defined in this paper. Figure 11 shows the evolution of the accumulated health sufficiency, along the pandemic in each of the considered international countries.

As it is observed in Figure 11, the aHSI has the same invariant performance for every country. That is, at the beginning of the pandemic it takes values close to zero as a lot of cases are suddenly confirmed and just a few of them are solved. At the end of the curves most confirmed cases are solved, so that the the aHSI is close to 100%. This invariant performance of the aHSI for all territories is the key to allowing a fair comparison among different countries with regards to the evolution of the pandemic. As already mentioned, the indicator just needs a sample to be calculated, which is the current case within the COVID-19 pandemic. While the available data do not correspond to the complete population of confirmed cases, it can be considered as a sample of such a population and, therefore, useful to build the indicator.

## 5. Discussion

The proposed indicators, related to the added pressure on a health system, have proved to be useful to monitor and compare the pandemic situation in different countries and regions. Using different sources of information, it has been possible to build and combine different indicators and variables to monitor health sufficiency at local, national and international levels.

In the local case, a predictive model for hospital occupancy has been built using these indicators together with other relevant exogenous variables from complementary sources of information. Through the proposed model, it is possible to predict in advance the requirements of hospital beds and ICU beds, allowing a more efficient organization of the resources of the hospital.

In the particular case of Spain, this indicator enables to really understand where the performance of Spain is in the global context of the pandemic. This is not only a merely indicative exercise, but also a methodology to define the right moment to relax the containment measures that are having such an impact on the economy, employment and quality of life of the citizens of the country. This comparison also permit knowing at what point other countries have begun to de-escalate the containment measures and the impact on the evolution of the pandemic in their territories.

The proposed Health Sufficiency Indicator, HSI, is a particularly useful tool for defining and monitoring the best public policies for each territory. The progressive exit from confinement not only allows a de-escalation that is more adjusted to the reality of each local region, but also the experience of the territories in the most advanced stages of de-confinement help to analyse the impact of the different measures on the evolution of the pandemic. This analysis will be especially useful when deciding whether the exit phases from confinement can be advanced or delayed in each territory. Actually, this is not a trivial issue. Carrying out a simultaneous and homogeneous de-escalation throughout the country implies putting the health system at risk in the territories with lower values of the indicators and unnecessarily delaying the return to economic activity in those regions that have already exceeded the security indexes. The HSI is a very useful tool for determining which regions have the capacity to react successfully to a resurgence and, therefore, can move more quickly towards de-confinement and thus avoid having their economy paralysed unnecessarily.

In addition, based on the daily and accumulated versions of the HSI indicators, it is possible to set different scenarios for the evolution of the pandemic. Low HSI values are to be expected in the early stages of a pandemic. By means of appropriate measures (such as confinement of the population), the aim is to increase these HSI values to stable levels of close to 100% for the aHSI and around 1 for the dHSI (simultaneously). It is at this point that it is considered that a pandemic is controlled by the health system and that, consequently, its incidence and alterations in the population and in the normal functioning of a health system are low and controlled (unlike in the initial phases in which situations of health insufficiency occur).

## 6. Conclusions

This paper has presented objective indicators on the added pressure on a health system due to a pandemic situation. These innovative indicators make it possible to monitor the capacity of health systems to respond to the needs of the population that generates a pandemic. In this way, it can be objectively verified whether the exceptional measures taken to deal with the pandemic have resulted in improvements and relief for the health system or, if not, it allows for the recognition of an adverse situation that requires stricter interventions.

In the experiments section, three levels for the combination of information has been considered. In the local case, a high number of exogenous variables have been used to predict the hospital wars bed occupancy and the ICU bed occupancy of a small region in Spain. In the country case, the hospital and ICU Potential Occupancy Ratio have been compared with the health sufficiency indicators in order to study the pandemic evolution in different regions of Spain. Finally, in the international case, the proposed health sufficiency indicators have been compared in the most affected countries.

The proposed indicators have demonstrated their ability to monitor the sufficiency of the health system and the behavior of the pandemic in different regions and countries, being useful for decision making at different levels.

Future work includes the development of a Pandemic Vulnerability Index that will operate as a real-time alert system monitoring emerging pandemics and allowing health managers to anticipate specific contingency plans based on objective and reliable data. A non-linear version of the dHSI following a logistic function would be also of great interest in an advanced version of the present study.

## Data Availability

Data supporting reported results can be found, in http://www.datasciencelab.es/research/projects/covid19/reports/covid19-report.html (accessed on 17 May 2021).

## Abbreviations

The following abbreviations are used in this manuscript:

aHSI	Accumulated Health Sufficiency Indicator
CFR	Case Fatality Rate
dHSI	Daily Health Sufficiency Indicator
hPOR	Potential Occupancy Ratio
HSI	Health Sufficiency Indicator
icuPOR	ICU Potential Occupancy Ratio
ICUs	Intensive Care Units
IFR	Infection Fatality Rate
POR	Hospital Potential Occupancy Ratio
